# Integrative multi-omics dissection identifies ACO2, KLF5, and IMP4 as central regulators of the mitochondrial–immune axis in ulcerative colitis

**DOI:** 10.3389/fimmu.2026.1746810

**Published:** 2026-03-27

**Authors:** Yuanyuan Tian, Xiaori Qin, Shibing Li, Jiao Wang, Cheng Lan

**Affiliations:** 1 Department of Gastroenterology, Hainan General Hospital, Hainan Affiliated Hospital of Hainan Medical University, Haikou, China; 2 Department of Pediatric Surgery, Hainan General Hospital, Hainan Affiliated Hospital of Hainan Medical University, Haikou, China; 3 Department of Infectious Diseases, Hainan General Hospital, Hainan Affiliated Hospital of Hainan Medical University, Haikou, China

**Keywords:** ACO2, immune–metabolic regulation, IMP4, KLF5, ulcerative colitis

## Abstract

**Background:**

Ulcerative colitis (UC) is a chronic relapsing inflammatory bowel disease characterized by persistent mucosal inflammation and epithelial barrier disruption. Emerging evidence suggests that metabolic reprogramming plays a pivotal role in regulating immune responses and epithelial homeostasis in UC. However, the key metabolic–immune regulatory genes and their cellular mechanisms remain poorly defined.

**Methods:**

We integrated publicly available genome-wide association study (GWAS) summary statistics for UC (n = 394,626), along with expression quantitative trait loci (eQTL) resources and multiple independent bulk transcriptomic datasets (total n = 215 cases and 134 controls). Summary-based Mendelian randomization (SMR), genome-wide Mendelian randomization (MR), and transcriptomic analyses were performed to systematically identify causal genes associated with UC. Cross-validation was conducted using immune infiltration analyses and single-cell RNA sequencing (scRNA-seq) datasets from human UC colonic tissues (n = 18 cases and 12 controls), as well as a dextran sulfate sodium (DSS)-induced murine colitis model. Gene set enrichment and network analyses were applied to explore potential metabolic and immune pathways.

**Results:**

Through integrative multi-omics analysis, we identified ACO2, KLF5, IMP4, and AGPS as key hub genes linking mitochondrial metabolism with immune regulation in UC. Among them, ACO2, KLF5, and IMP4 were consistently downregulated in UC tissues and negatively correlated with macrophage and dendritic cell infiltration. Although AGPS did not show consistent transcriptional changes across UC datasets, it may still contribute to lipid remodeling based on its metabolic function. Single-cell analyses revealed that ACO2 and KLF5 were primarily expressed in macrophage populations and markedly reduced in inflamed colonic regions, while IMP4 exhibited context- and cell-type–specific dynamics. In the DSS mouse model, Aco2 and Klf5 expression decreased progressively with disease severity, accompanied by metabolic pathway enrichment in oxidative phosphorylation and glycolysis.

**Conclusion:**

Our findings reveal a set of metabolic–immune regulatory genes that orchestrate mitochondrial function, epithelial integrity, and immune activation in UC. The integration of genetic, transcriptomic, and single-cell data highlights ACO2, KLF5, and IMP4 as promising biomarkers and potential therapeutic targets, offering novel insights into the immunometabolism mechanisms driving UC pathogenesis.

## 1 Introduction

Ulcerative colitis (UC) is a persistent and recurrent form of inflammatory bowel disease marked by continuous inflammation of the colonic mucosa. Clinically, patients typically present with abdominal pain and diarrhea, and disease progression may lead to severe complications such as colorectal cancer or toxic megacolon (1). Although the introduction of biological therapies has improved disease management, a considerable proportion of patients still exhibit poor or unstable responses, highlighting the substantial heterogeneity in disease pathogenesis and treatment outcomes (2). This clinical variability highlights the multifactorial nature of UC, where genetic risk, immune imbalance, and epithelial barrier dysfunction converge (3). In addition, accumulating evidence indicates that metabolic dysregulation contributes to UC pathogenesis. Mitochondrial dysfunction and altered oxidative phosphorylation have been linked to epithelial barrier impairment, while lipid remodeling and immune metabolic reprogramming sustain aberrant inflammatory responses (4, 5). Nevertheless, the key genes connecting genetic susceptibility to immune–metabolic remodeling in UC remain poorly defined.

Multi-omics integration offers new opportunities to dissect disease mechanisms beyond the scope of conventional GWAS or transcriptomic approaches. Genome-wide association studies (GWAS) have successfully identified hundreds of UC-associated loci (6). However, the functional interpretation of these variants remains challenging, as most reside in non-coding regions. Expression quantitative trait loci (eQTL) mapping and Mendelian randomization (MR) offer a framework to bridge this gap by linking genetic variants to gene expression and downstream phenotypic consequences, thereby enabling causal inference of candidate genes (7). When coupled with Summary data-based Mendelian Randomization (SMR) and transcriptomic validation, this integrative strategy has proven effective in uncovering functional mediators and cell-type–specific drivers in complex immune diseases such as rheumatoid arthritis, schizophrenia, and neurodevelopmental disorders (8–10). In parallel, data-driven machine learning approaches have revolutionized the analysis of high-dimensional omics data, enabling the discovery of consistent biomarkers and elucidation of nonlinear molecular interactions underlying disease variability (11). Integrating these computational strategies enhances both the robustness and interpretability of causal gene inference, particularly in complex immune-mediated disorders.

Recent innovations in single-cell and spatial transcriptomics have provided unprecedented resolution in dissecting ulcerative colitis pathogenesis. These investigations reveal diverse immune and epithelial subpopulations and elaborate crosstalk between macrophages, T cells and neutrophils that underlies sustained inflammation and epithelial damage (12, 13). Notably, several cell types—including pathogenic B cells, CD8^+^ effector T cells, and regenerating epithelial subsets—have been implicated in shaping disease activity and therapeutic response (13–15). However, despite these advances, how genetically driven transcriptional programs reshape cellular metabolism to influence immune activation and epithelial integrity in UC is still poorly understood. In particular, how genetically determined transcriptional programs influence metabolism and immune regulation in UC is still poorly understood.

**Figure 1:**
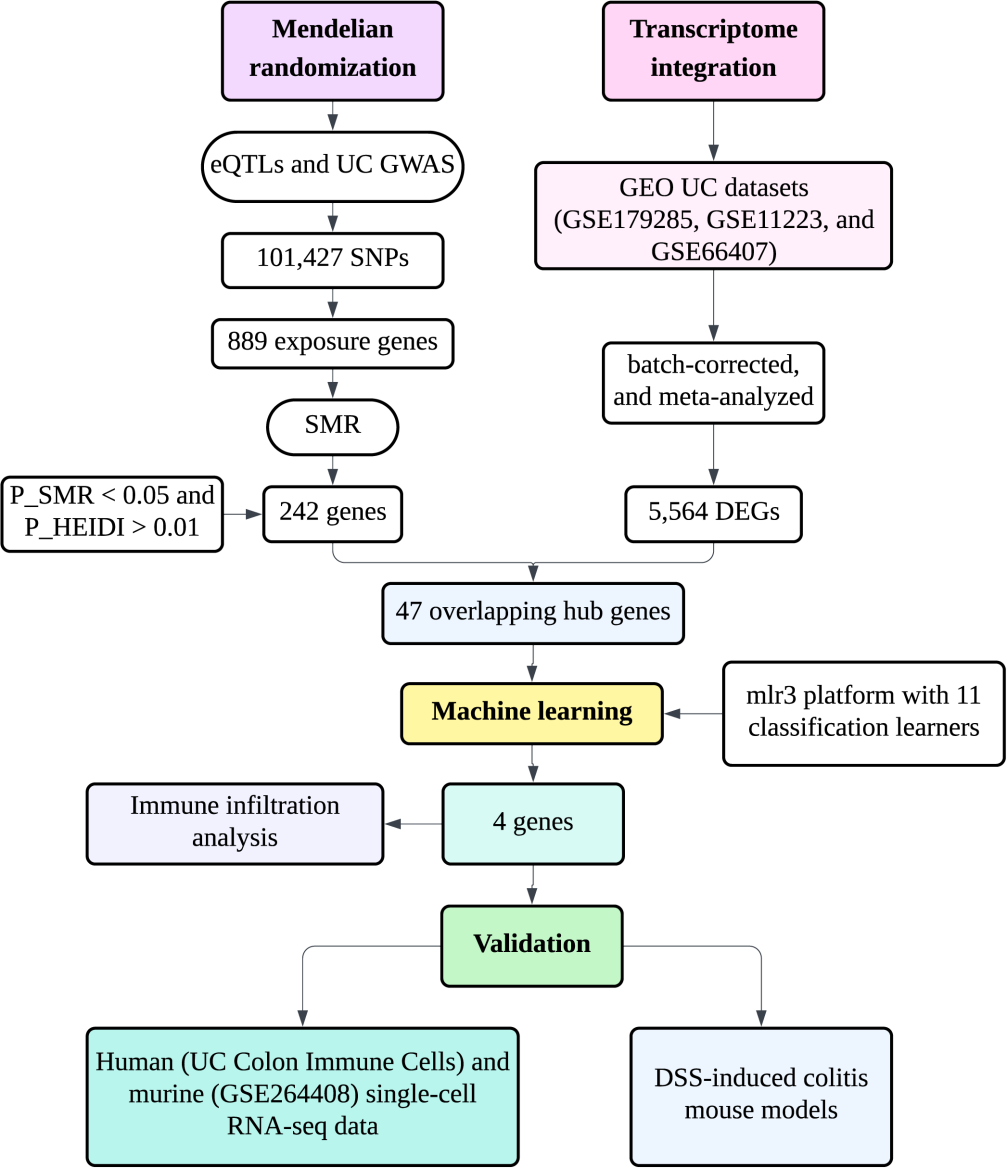
Overview of the multi-omics strategy integrating causal inference (MR/SMR), transcriptome meta-analysis, machine learning, immune infiltration profiling, single-cell validation, and experimental confirmation to identify mitochondrial–immune regulatory genes in UC.

To address these gaps, we implemented an integrative multi-omics framework that connects genetic causality, transcriptional regulation, and cellular specificity in UC. We first leveraged large-scale eQTL and GWAS datasets to identify genetically regulated genes associated with UC risk through Mendelian randomization and summary-based co-localization analyses. These candidate genes were further validated across multiple bulk transcriptomic cohorts and refined by co-expression network and machine learning–based diagnostic modeling. Finally, we combined immune infiltration profiling and single-cell transcriptomic validation to uncover the cellular origins and potential roles of key hub genes in immune–metabolic remodeling (**Figure 1**).

## 2 Materials and methods

### 2.1 Study design and analytical workflow

A multi-omics integrative strategy was employed to identify and validate key regulatory genes linking mitochondrial metabolism and immune remodeling in UC (**Figure 1**). The overall workflow included: (1) Causal inference: Mendelian randomization (MR) and summary-data–based Mendelian randomization (SMR) were conducted by integrating UC GWAS (GCST90468152) and GTEx v10 eQTL data to identify potential causal genes associated with UC susceptibility.(2) Transcriptome integration: Gene expression profiles from three independent UC cohorts (GSE179285, GSE11223, and GSE66407) were normalized, batch-corrected, and meta-analyzed to validate the differential expression of candidate genes. (3) Machine learning diagnostic modeling: Candidate genes were further screened using eleven machine learning algorithms to identify robust hub genes with diagnostic potential for UC. (4) Immune infiltration analysis: The proportions of immune and stromal cell populations were estimated using xCell, EPIC, quanTIseq, and MCP-counter algorithms, and their correlations with hub gene expression were evaluated. (5) Single-cell validation: Publicly available single-cell RNA-seq datasets from human (UC Colon Immune Cells) and murine (GSE264408) colonic tissues were analyzed to map gene expression to distinct immune and epithelial subsets across inflammation states. (6) Experimental validation and functional annotation: DSS-induced colitis mouse models were used to verify hub gene expression by qPCR.

### 2.2 GWAS and expression quantitative trait loci data sources

The primary GWAS summary statistics for UC were obtained from the GWAS Catalog (GCST90468152) (16). Expression quantitative trait loci (eQTL) data from the sigmoid colon were derived from the GTEx v10 database (17). Sigmoid colon eQTLs were selected because this region exhibits the strongest and most consistent inflammatory transcriptional alterations in UC. SNPs with minor allele frequency (MAF) < 0.01 were excluded, and all datasets were harmonized to the GRCh38 reference genome.

### 2.3 Mendelian randomization and SMR analysis

Causal inference between genetically predicted gene expression and UC risk was evaluated using two-sample Mendelian randomization (MR). Independent instrumental variables were selected based on LD clumping (r² < 0.01, 10 Mb window). Inverse variance weighted (IVW), Weighted median, MR Egger and Weighted mode were implemented using the TwoSampleMR R package (18). To identify and account for possible horizontal pleiotropy or outlier influences, we performed the MR-PRESSO global test followed by its outlier adjustment procedure, thereby increasing the robustness of causal estimation (19).

Summary-data–based Mendelian randomization (SMR) and SMR locus plot were implemented to integrate GWAS and eQTL summary statistics for the identification of genes exhibiting potential causal or pleiotropic associations (8). Allele harmonization was performed to ensure alignment of effect alleles between eQTL and GWAS summary statistics prior to SMR analysis. To distinguish true pleiotropy from confounding by linkage, heterogeneity in dependent instruments (HEIDI) testing was applied (P < 0.05). Genes fulfilling both SMR and HEIDI thresholds were considered to have plausible causal relevance.

### 2.4 Transcriptome data acquisition and preprocessing

Three independent ulcerative colitis (UC) transcriptome datasets — GSE179285, GSE11223, and GSE66407 — were obtained from the Gene Expression Omnibus (GEO, https://www.ncbi.nlm.nih.gov/geo/). Inclusion criteria required that datasets be derived from human sigmoid colon tissues and contain both UC and healthy control samples. Dataset GSE179285 (platform GPL6480) includes RNA-seq data from 22 UC and 11 control biopsies (20). GSE11223 (GPL1708) includes 32 UC and 24 control samples (21), and GSE66407 (GPL19833) comprises 161 UC and 99 control specimens. All expression matrices were log_2_-normalized, and batch effects were minimized with the ComBat method (sva R package). The NetworkAnalyst platform was used for meta-integration procedures (22).

### 2.5 Immune cell infiltration and correlation analysis

The immune microenvironment of ulcerative colitis was profiled by estimating immune cell infiltration using xCell, EPIC, quanTIseq, and MCP-counter algorithms from immunedeconv R package (23). Differences in inferred immune composition between UC and control samples were determined by Wilcoxon rank-sum tests with FDR correction. Correlations between core gene expression and immune cell abundance were calculated using Spearman’s method, and statistically significant correlations (|r| > 0.3, P < 0.05) were illustrated as heatmaps generated with the pheatmap R package.

### 2.6 Machine learning analysis

To examine the diagnostic utility of the hub genes, we constructed a series of supervised machine learning (ML) classifiers using the mlr3 R package (24).The analytical pipeline consisted of five sequential steps:(a) defining ML tasks and selecting learners based on normalized gene expression profiles; (b) Gene expression profiles from GSE66407 and GSE179285 were merged and used as the training cohort, while GSE11223 served as the independent validation cohort. (c) implementing 10-fold cross-validation within the training data to mitigate overfitting and ensure internal reliability; (d) training each learner and generating corresponding predictive outcomes; and (e) selecting the best-performing classifier based on both internal CV and external validation performance.

A total of eleven supervised algorithms frequently applied in biomedical data classification were tested, including elastic net–regularized generalized linear model (glmnet), random tree, support vector machine (SVM), multinomial logistic regression, single-layer neural network (nnet), decision tree (rpart), naïve Bayes, random forest, k-nearest neighbor (kknn), gradient boosting (lightgbm), and extreme gradient boosting (xgboost). Model performance was evaluated based on ROC and AUC metrics, complemented by calibration curve analyses to assess discrimination and prediction consistency.

### 2.7 Single-cell RNA sequencing analysis

Single-cell transcriptome data from the UC Colon Immune Cells dataset (Broad DUOS), comprising 366,650 cells from 30 individuals (18 UC patients and 12 healthy controls) (25). Single-cell analysis was restricted to immune cell populations. The murine dataset GSE264408 (26) was analyzed, which includes colon tissues from 3 healthy mice, 3 mice with DSS-induced acute colitis, and 4 mice with DSS-induced chronic colitis (26). Raw expression matrices were processed using Seurat (27). Quality control was performed by excluding cells with <200 or >5000 detected genes and mitochondrial gene content >15%. Normalization and dimensionality reduction were conducted using SCTransform and principal component analysis.

Cell clusters were annotated based on canonical marker genes and metadata from the original publications. Differential gene expression across clusters and colitis states was assessed using the Wilcoxon rank-sum test (adjusted P<0.05). For functional characterization of hub genes, differential expression analysis was first performed across all immune cell clusters. For each hub gene, correlation analysis was performed between the expression level of the hub gene and all differentially expressed genes (DEGs) across the UC samples using Spearman correlation analysis. Genes showing significant correlations with the hub gene (|r| > 0.3 and P < 0.05) were selected to construct gene subsets for each hub gene. These gene lists were subsequently subjected to Gene Ontology (GO) enrichment analysis using the clusterProfiler R package (28). Pathways with adjusted P < 0.05 were considered statistically significant. All spearman correlation coefficients were calculated between average hub gene expression per sample and inferred immune cell proportions. Correlations with |r| > 0.3 and adjusted P < 0.05 were retained for network visualization using the igraph R package. Positive and negative correlations were indicated by red and blue edges, respectively.

### 2.8 Animal model and qPCR validation

A murine DSS-induced colitis model was established in C57BL/6J mice (6–8 weeks old). Mice were administered 2.5% DSS for 5 days followed by regular water for 2 days, and mice were sacrificed on day 7. Age- and sex-matched control mice received regular drinking water throughout the entire experimental period. At the experimental endpoint, animals were euthanized by gradual-fill CO_2_ inhalation at a displacement rate of 20–30% chamber volume per minute, followed by cervical dislocation to ensure death. Colonic tissues were collected for RNA extraction using TRIzol (Invitrogen), and quantitative PCR was performed using SYBR green kit (Toyobo, Osaka, Japan). Relative gene expression was calculated by the 2^^–ΔΔCt^ method using Gapdh as a reference. Primer pairs: 5’- TCTCCACACCTATGGTGCAA -3’ and 5’- CAAGAAACAGGGGAGCTGAG -3’ (Gapdh); 5’- TGAGTACATCCGATATGACCTGC -3’ and 5’- GAGAGTAAGAGGCCGGTTCAA -3’ (Aco2); 5’- CCGGAGACGATCTGAAACACG -3’ and 5’- GTTGATGCTGTAAGGTATGCCT -3’ (Klf5); 5’- AAGGAGCGAGTCAAGCGTG -3’ and 5’- CACCAGCATCATCGAACTCCA -3’ (Imp4); 5’- CTGGAGAAGATAACGGTCAGAGG -3’ and 5’- CCCCTTTCTTTGCATTCCCTT -3’ (Agps); All animal procedures were approved by the institutional ethics committee.

### 2.9 Statistical analysis

All statistical analyses were performed in R (v4.3.0). Data were expressed as mean ± standard error (SEM). Two-group comparisons were analyzed using Student’s t-test or Wilcoxon test, as appropriate. Multiple comparisons were corrected by the Benjamini–Hochberg method. Results were considered statistically significant at P < 0.05 unless otherwise indicated.

## 3 Results

### 3.1 Identification of genetically regulated ulcerative colitis–associated genes through integrative MR, SMR, and transcriptomic analyses

**Figure 2:**
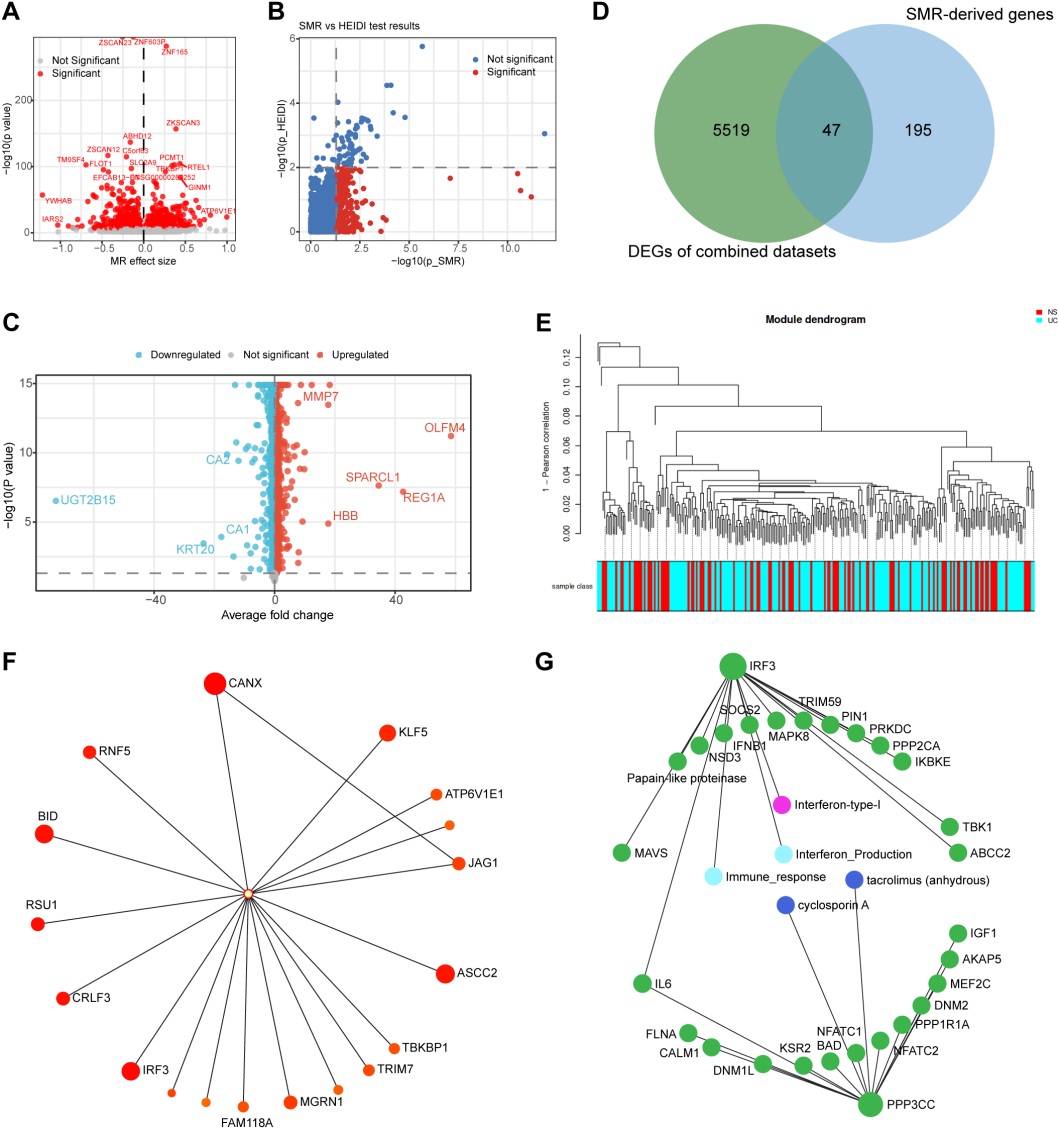
Identification of genetically regulated ulcerative colitis–associated genes through integrative MR, SMR, and transcriptomic analyses. **(A)** Manhattan plot showing causal associations between gene expression and UC risk identified through two-sample Mendelian randomization. **(B)** SMR results highlighting 242 genes passing both SMR significance (p_SMR < 0.05) and co-localization criteria (p_HEIDI > 0.01). **(C)** Volcano plot of DEGs identified from integrated transcriptomic meta-analysis across three UC datasets using the ExpressAnalyst platform. **(D)** Venn diagram illustrating 47 overlapping hub genes shared between SMR-derived and combined datasets-derived DEGs. **(E)** Module–trait relationship heatmap derived from automated WGCNA, showing significant modules correlated with UC. **(F)** Co-expression network visualization of hub genes within the key UC-associated modules and **(G)** KEGG pathway.

To identify genes with a potential causal relationship with ulcerative colitis (UC), we first performed a MR analysis using eQTLs from the sigmoid colon as exposure variables and GWAS summary statistics for UC (GCST90468152) as outcome data. After harmonization and quality control, a total of 101,427 SNPs were included as instrumental variables. Following heterogeneity and horizontal pleiotropy testing, a total of 889 exposure genes showed genome-wide significant causal associations with UC (p < 5 × 10^−8^). These results reflect a broad landscape of genetically regulated expression effects potentially involved in UC pathogenesis. (**Figure 2A**) (**Supplementary Tables 1**, **2**).

To further prioritize causal candidates, we next conducted SMR analysis. Using stringent thresholds (p_SMR < 0.05 and p_HEIDI > 0.01), 242 genes met both MR and co-localization criteria (**Figure 2B**) (**Supplementary Table 3**). In parallel, an independent transcriptomic meta-analysis was performed using three ulcerative colitis datasets integrated through the ExpressAnalyst platform. Across the combined datasets, 5,564 differentially expressed genes (DEGs) were identified (adjusted p < 0.05), delineating a comprehensive transcriptional signature of UC mucosal inflammation (**Figure 2C**). Intersecting these DEGs with the 242 SMR-derived genes yielded 47 overlapping hub genes (**Figure 2D**), representing transcriptionally and genetically convergent UC-associated candidates.

Co-expression network analysis using automated WGCNA (**Figure 2E**) identified distinct gene modules significantly associated with ulcerative colitis status. These 47 hub genes were clustered within highly interconnected modules (**Figure 2F**), indicating coordinated transcriptional regulation. Functional enrichment of these modules (**Figure 2G**) highlighted biological processes related to immune activation, interferon signaling, and cyclosporin A response.

### 3.2 Machine learning–based identification and validation of diagnostic signatures in UC

To prioritize candidate genes with diagnostic potential and to refine the SMR- and transcriptome-derived hub gene set, we implemented a supervised machine learning framework using the mlr3 platform. 11 classification learners were systematically evaluated, including regularized generalized linear models, Random Forest, eXtreme Gradient Boosting (XGBoost), Support Vector Machines, and other standard classifiers (see Methods). The datasets GSE66407 and GSE11223 were merged to form the training cohort, while GSE179285 served as an independent validation cohort. For each learner, hyperparameter optimization was conducted using grid or random search combined with 10-fold cross-validation to minimize overfitting and ensure model generalizability.

**Figure 3:**
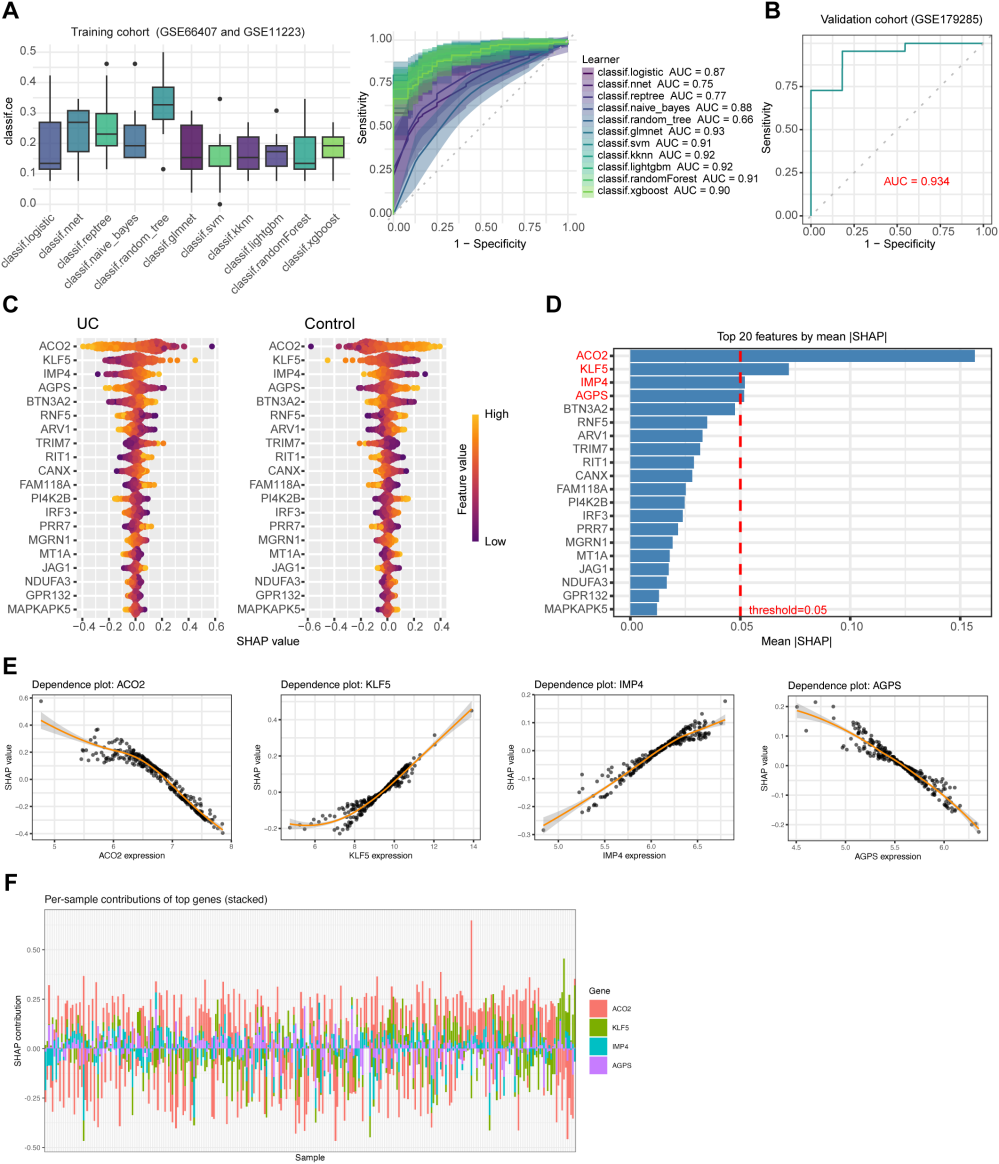
Machine learning–based identification and validation of diagnostic signatures in ulcerative colitis. **(A)** Comparative performance of 11 machine learning classifiers evaluated on the training data using 10-fold cross-validation. The elastic-net regularized generalized linear model (glmnet) demonstrated the best overall AUC and stability. **(B)** ROC curves showing the performance of the glmnet model in both the training and independent validation datasets. **(C)** Mean SHAP importance plot displaying the top 20 genes contributing to UC classification; four genes (ACO2, KLF5, IMP4, and AGPS) exceeded the SHAP threshold (mean importance > 0.05). **(D)** SHAP summary plot illustrating per-gene feature effects on UC prediction across all samples. **(E)** Scatter plots of SHAP values versus normalized gene expression for ACO2, KLF5, IMP4, and AGPS. **(F)** Per-sample SHAP dependence plots showing the contribution of the four hub genes to the glmnet model predictions.

Among all models, the elastic-net regularized generalized linear model implemented via glmnet (generalized linear model with elastic-net penalty) demonstrated the most stable and accurate classification performance (AUC = 0.932, **Figure 3A**). After hyperparameter tuning, the glmnet model achieved high discriminative ability in the training data and retained robust performance in the validation dataset, with AUC values exceeding 0.934 (**Figure 3B**). To interpret the predictive features, we extracted the top 20 genes ranked by absolute model coefficients (feature importance) from the final glmnet model. Four genes—ACO2 (aconitase 2), KLF5 (Krüppel-like factor 5), IMP4 (IMP U3 small nucleolar ribonucleoprotein 4), and AGPS (alkylglycerone phosphate synthase)—consistently ranked among the top predictive features across cross-validation folds (mean SHAP importance > 0.05; **Figure 3C**). ACO2 is a key enzyme of the tricarboxylic acid (TCA) cycle involved in mitochondrial energy metabolism; KLF5 is a transcription factor implicated in epithelial proliferation and inflammatory signaling; IMP4 participates in ribosomal RNA processing and cellular growth regulation; and AGPS is a peroxisomal enzyme essential for ether lipid biosynthesis. The SHAP summary plot further visualized the contribution of each feature to UC prediction across samples (**Figure 3D**). Scatter plots of SHAP values versus gene expression (**Figure 3E**) showed strong relationships for each gene, indicating that changes in expression meaningfully and consistently influenced model predictions. At the sample level, per-sample SHAP contribution plots (**Figure 3F**) illustrated how each of the four genes contributed to individual predictions across cases and controls.

### 3.3 Causal and expression analyses confirm the functional relevance of ACO2, KLF5, IMP4, and AGPS in UC

**Figure 4:**
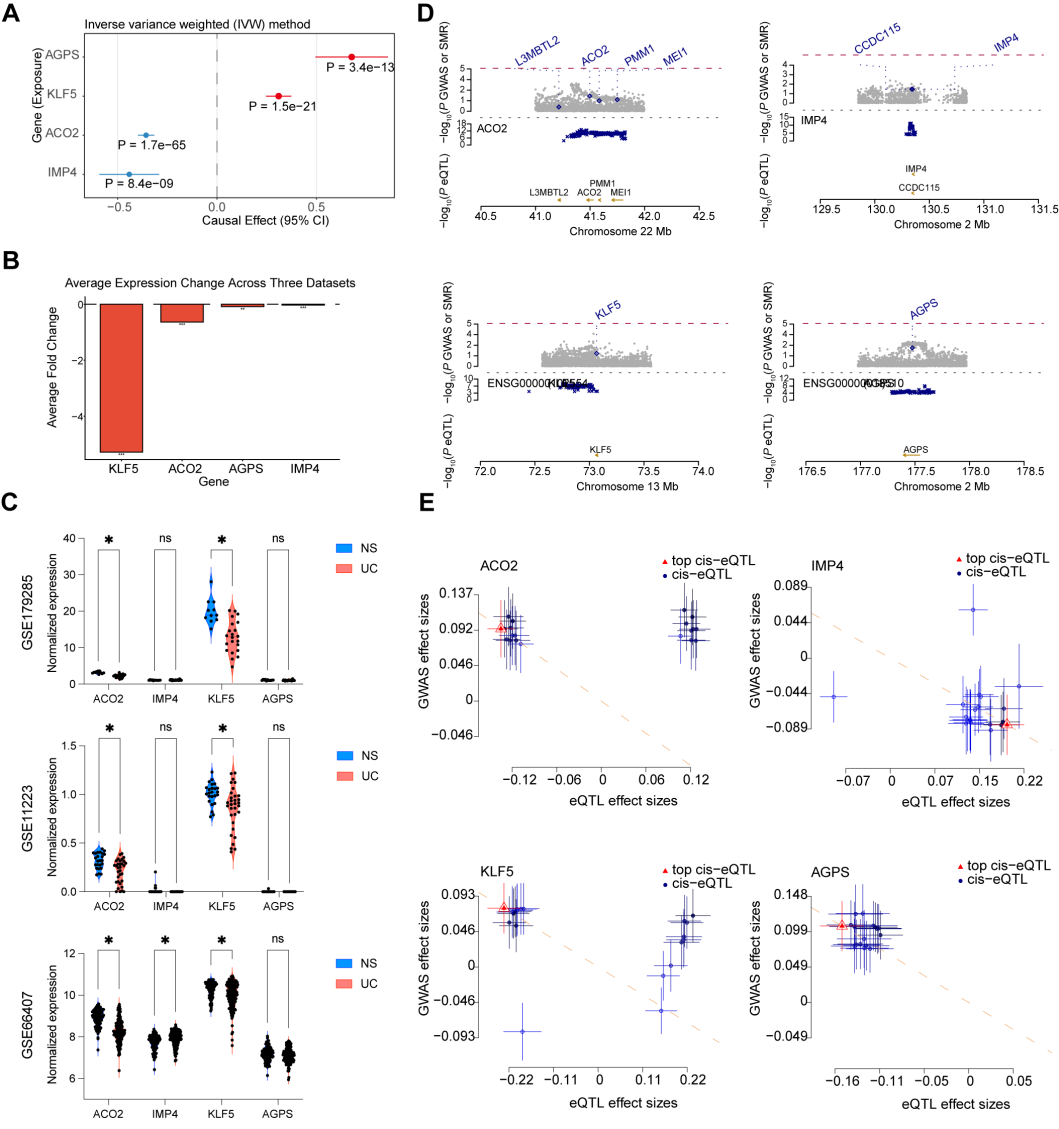
Causal and expression analyses confirm the functional relevance of ACO2, KLF5, IMP4, and AGPS in UC. **(A)** Forest plot showing causal effects of ACO2, KLF5, IMP4, and AGPS on UC from Mendelian randomization (MR) analysis. **(B)** Average log_2_ fold change of the four genes across three meta-transcriptomic datasets. **(C)** Comparison of normalized gene expression levels between UC and normal samples across three independent transcriptomic cohorts (GSE179285, GSE11223, and GSE66407). Expression values correspond to dataset-specific normalized expression as provided in the original studies. **(D)** SMR locus plots showing the regional colocalization of GWAS and eQTL signals for each gene. **(E)** SMR effect plots illustrating the concordant effects between GWAS and eQTL associations. (*p < 0.05).

To further validate the causal roles of the identified candidate genes, we conducted a series of Mendelian randomization (MR) and expression analyses. The MR-based causal inference demonstrated that AGPS and KLF5 exhibited positive causal effects on UC risk, whereas ACO2 and IMP4 showed negative effects (**Figure 4A**). Meta-analysis of three independent transcriptomic datasets consistently revealed reduced average fold changes for all four genes in ulcerative colitis compared to normal tissues (**Figure 4B**). Differential expression analyses further confirmed that ACO2 and KLF5 were significantly downregulated in UC across all datasets. IMP4 was significantly upregulated in GSE66407, whereas it showed a decreasing trend in GSE179285 and GSE11223, although the differences did not reach statistical significance (**Figure 4C**). SMR locus plots showed clear colocalization between GWAS and eQTL signals for the four genes, supporting shared causal variants influencing both gene expression and UC susceptibility (**Figure 4D**). SMR effect plots indicated consistent effect directions between eQTL and GWAS data, reinforcing the pleiotropic relationship and causal relevance of these genes in UC pathogenesis (**Figure 4E**). HEIDI tests supported pleiotropic associations for KLF5 (p_HEIDI = 0.1209), IMP4 (p_HEIDI = 0.5702), and AGPS (p_HEIDI = 0.8477), suggesting shared causal variants underlying both gene expression and UC susceptibility. For ACO2, although the SMR association was significant, the HEIDI test indicated potential heterogeneity (p_HEIDI = 0.0102), suggesting that this locus may have a more complex LD structure and should be interpreted with caution.

### 3.4 Immune cell association and infiltration analysis of the four hub genes

**Figure 5:**
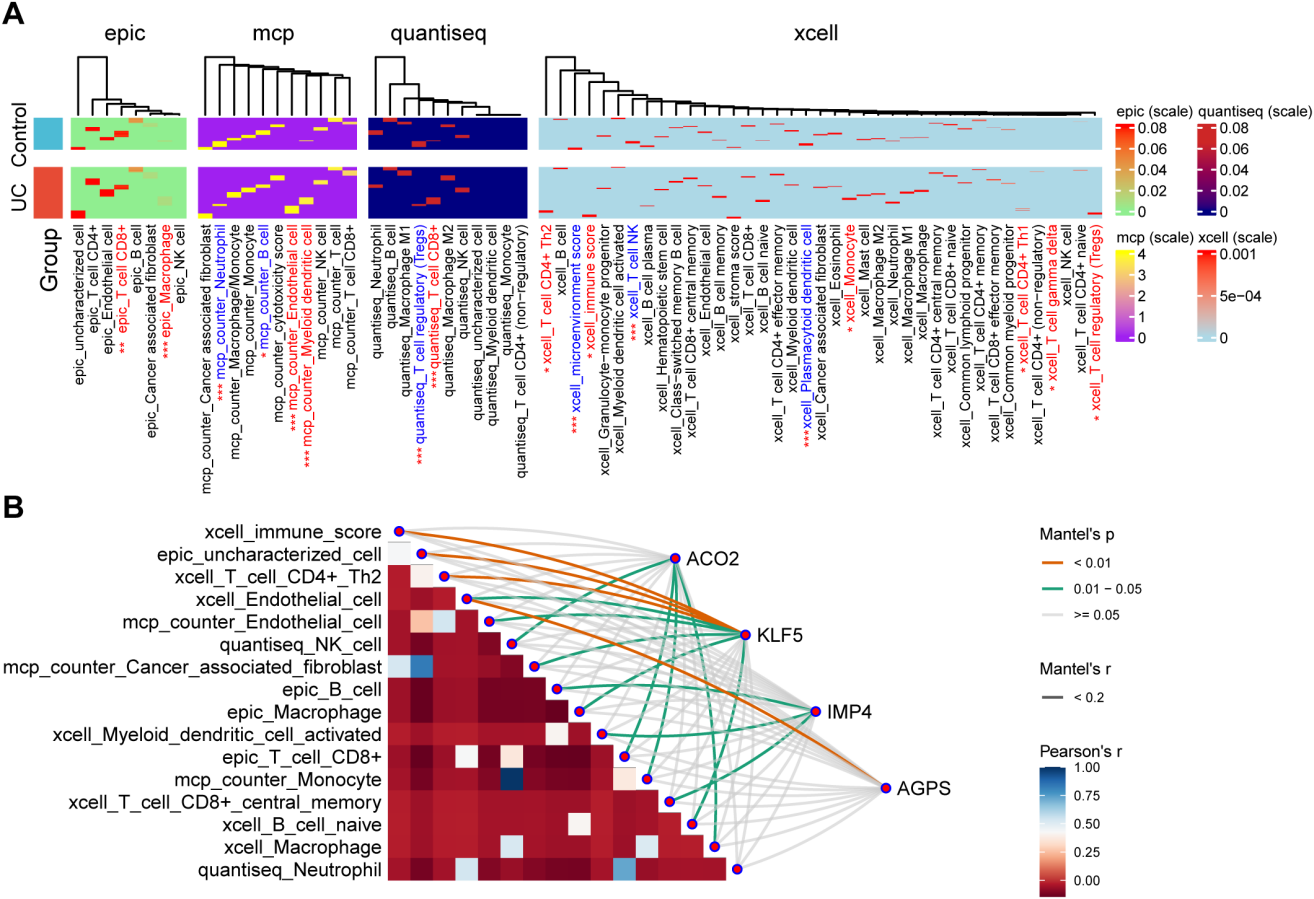
Immune cell association and infiltration analysis of the four hub genes. **(A)** Differential immune infiltration analysis between UC and control colonic mucosa. The heatmap displays the abundance of immune and stromal cell populations estimated by multiple deconvolution algorithms (EPIC, quanTIseq, MCP-counter, xCell). Cells with significant abundance differences (FDR < 0.05) are labeled; red indicates enrichment in UC (diff > 0), and blue indicates depletion (diff < 0). (***p < 0.001, **p < 0.01, *p < 0.05). **(B)** Correlation heatmap showing associations between the expression levels of ACO2, KLF5, IMP4, and AGPS and the estimated immune cell fractions. Positive correlations are indicated in red and negative correlations in blue.

To further characterize the immunological context of the four hub genes, we investigated their associations with immune cell infiltration of the combined datasets. Using multiple immune deconvolution algorithms (EPIC, quantiseq, MCP-counter, and xCell), we quantified the relative abundance of diverse immune and stromal cell populations within the colonic mucosa. Differential infiltration analysis revealed 15 immune or stromal cell populations showing significant differences (FDR < 0.05) between UC and control tissues (**Figure 5A**). Notably, endothelial cells (Δ = +0.60, FDR = 9.25 × 10^−5^), myeloid dendritic cells (Δ = +0.42, FDR = 0.0034), and CD8^+^ T cells (Δ = +0.0082, FDR = 0.0034) were significantly enriched in UC mucosa, whereas neutrophils (Δ = –0.69, FDR = 9.25 × 10^−5^), regulatory T cells (Δ = –0.0131, FDR = 1.12 × 10^−4^), and B cells (Δ = –0.37, FDR = 0.0466) were markedly depleted. These findings delineate a remodeled immune microenvironment characterized by endothelial activation, increased antigen-presenting activity, and disruption of suppressive and humoral immune components.

To elucidate the immunological functions of the four hub genes, we performed correlation analyses between their expression levels and immune cell fractions across all datasets (**Figure 5B**). ACO2 expression positively correlated with several innate and adaptive immune populations, including NK cells, CD8^+^ T cells, monocytes, and macrophages. KLF5 exhibited positive correlations with Th2 cells, macrophages, and naïve B cells, suggesting involvement in type 2 immune polarization and macrophage differentiation. IMP4 was positively associated with B cells, activated myeloid dendritic cells, and central memory CD8^+^ T cells, indicating a potential link to antigen presentation and adaptive memory formation. AGPS expression correlated positively with endothelial cells, implying a role in vascular activation and tissue remodeling during inflammation.

Finally, the overall expression comparison of these four hub genes across three independent UC transcriptome datasets consistently demonstrated significant downregulation in UC mucosa relative to controls, with combined meta-analysis p < 0.01 for all four genes (**Supplementary Table 4**). The average fold changes were –0.64 for ACO2, –0.33 for KLF5, –0.03 for IMP4, and –0.09 for AGPS, supporting their consistent suppression in UC lesions.

### 3.5 Single-cell transcriptomic analysis reveals immune-cell–specific expression and correlations of key causal genes

**Figure 6:**
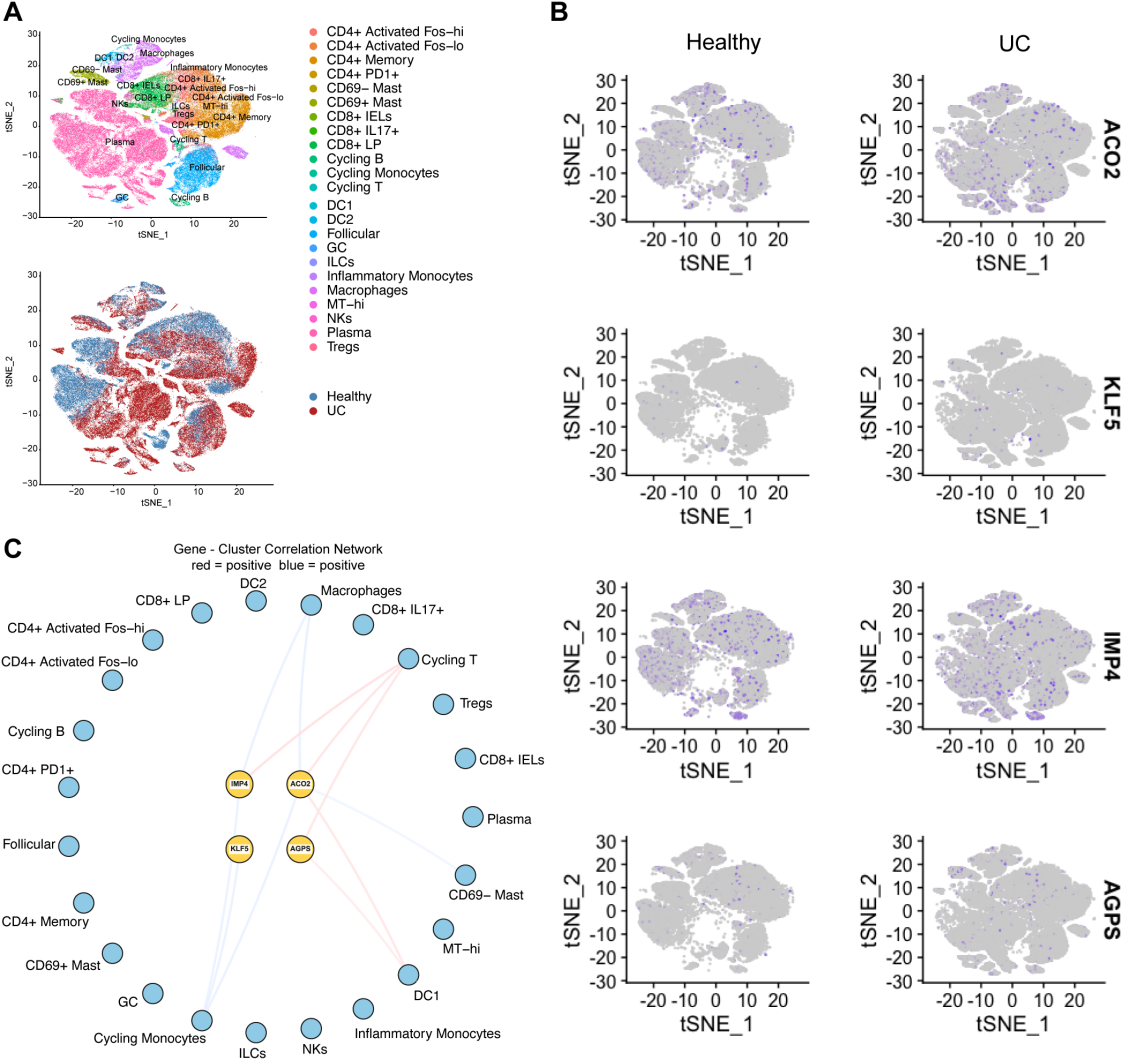
Single-cell transcriptomic analysis reveals immune-cell–specific expression and correlations of key causal genes. **(A)** t-SNE plots depicting the immune-cell clusters and the separation between healthy controls and UC samples. **(B)** Feature plots showing the expression distributions of ACO2, KLF5, IMP4, and AGPS across immune-cell subsets. **(C)** Correlation network illustrating associations between gene expression and immune-cell clusters.

**Figure 7:**
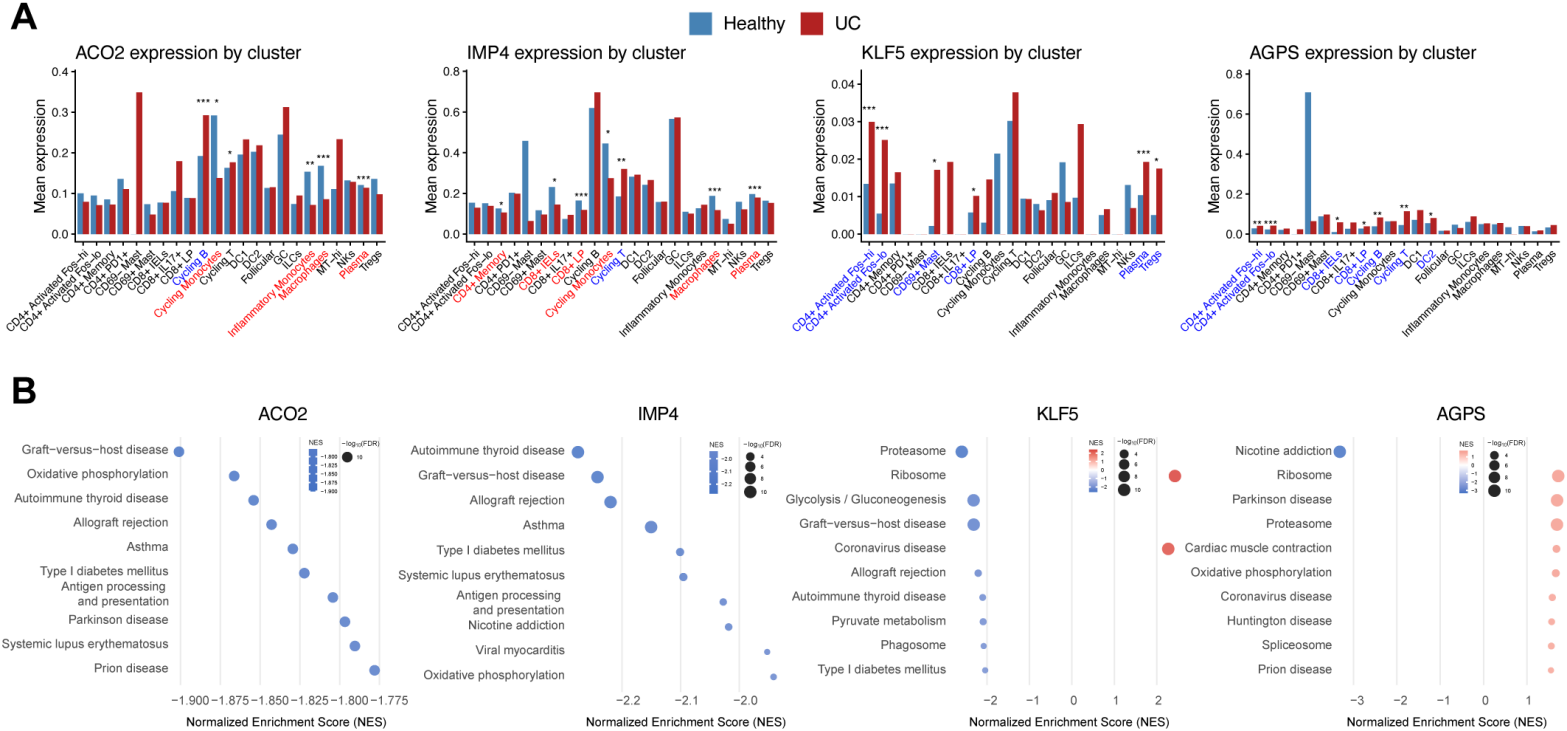
Differential expression and pathway enrichment analyses reveal immune-cell–specific functional alterations of the four causal genes. **(A)** Bar plots showing differential expression of ACO2, KLF5, IMP4, and AGPS between healthy and UC samples across immune-cell clusters. **(B)** Top enriched biological pathways associated with the four genes derived from enrichment analysis, illustrating their potential roles in metabolic regulation and immune activation in UC. (***p < 0.001, **p < 0.01, *p < 0.05).

To further characterize the cellular context of the identified genes, we analyzed a high-resolution single-cell transcriptomic dataset from colonic immune cells of patients with UC and healthy controls. The t-SNE visualization delineated 23 distinct immune cell clusters, showing clear segregation between ulcerative colitis (UC) and healthy samples (**Figure 6A**). Feature plot analyses revealed distinct spatial expression patterns of ACO2, KLF5, IMP4, and AGPS across immune-cell clusters. ACO2 and KLF5 were relatively enriched in immune cell populations, particularly macrophage and selected T-cell subsets, compared to other immune clusters (**Figure 6B**). Quantitative comparisons of gene expression between UC and healthy samples across clusters are presented in **Figure 7A**. Correlation network analysis further showed significant associations between hub gene expression and inferred immune cell proportions (|r| > 0.3, adjusted P < 0.05) (**Figure 6C**), suggesting that IMP4, ACO2, and AGPS were positively correlated with cycling T cells, whereas IMP4, KLF5, and ACO2 showed negative correlations with cycling monocytes. In addition, IMP4 and ACO2 were inversely correlated with macrophages (**Figure 6C**). These correlations suggest potential associations between mitochondrial gene expression and immune cell composition, pointing to a potential link between mitochondrial gene expression and immune cell remodeling in UC.

### 3.6 Differential expression and pathway enrichment analyses reveal immune-cell–specific functional alterations of the four causal genes

To further explore the immune-cell–specific transcriptional changes of the four causal genes, we compared their expression between UC and healthy samples across immune clusters. ACO2 and IMP4 showed significantly reduced expression in cycling monocytes, macrophages, and plasma cells (adjusted P < 0.05), whereas KLF5 and AGPS exhibited increased expression in CD4^+^ activated FOS-hi and CD8^+^ LP T-cell subsets (**Figure 7A**). These findings suggest potential suppression of mitochondrial and translational activity in myeloid compartments and enhanced transcriptional programs in effector T-cell subsets.

To explore the functional context of the hub genes, cluster-specific differential expression analysis was first performed across all immune cell populations. For each hub gene, differentially expressed genes significantly associated with its expression were extracted and used as input for functional enrichment analysis. The top enriched GO terms for each gene are presented in **Figure 7B**, including Antigen processing and presentation, Oxidative phosphorylation, and Glycolysis. These results indicate the involvement of both immune-related and metabolic pathways in UC.

### 3.7 Single-cell validation of dynamic expression and functional associations of four key genes in a murine DSS-induced colitis model

**Figure 8:**
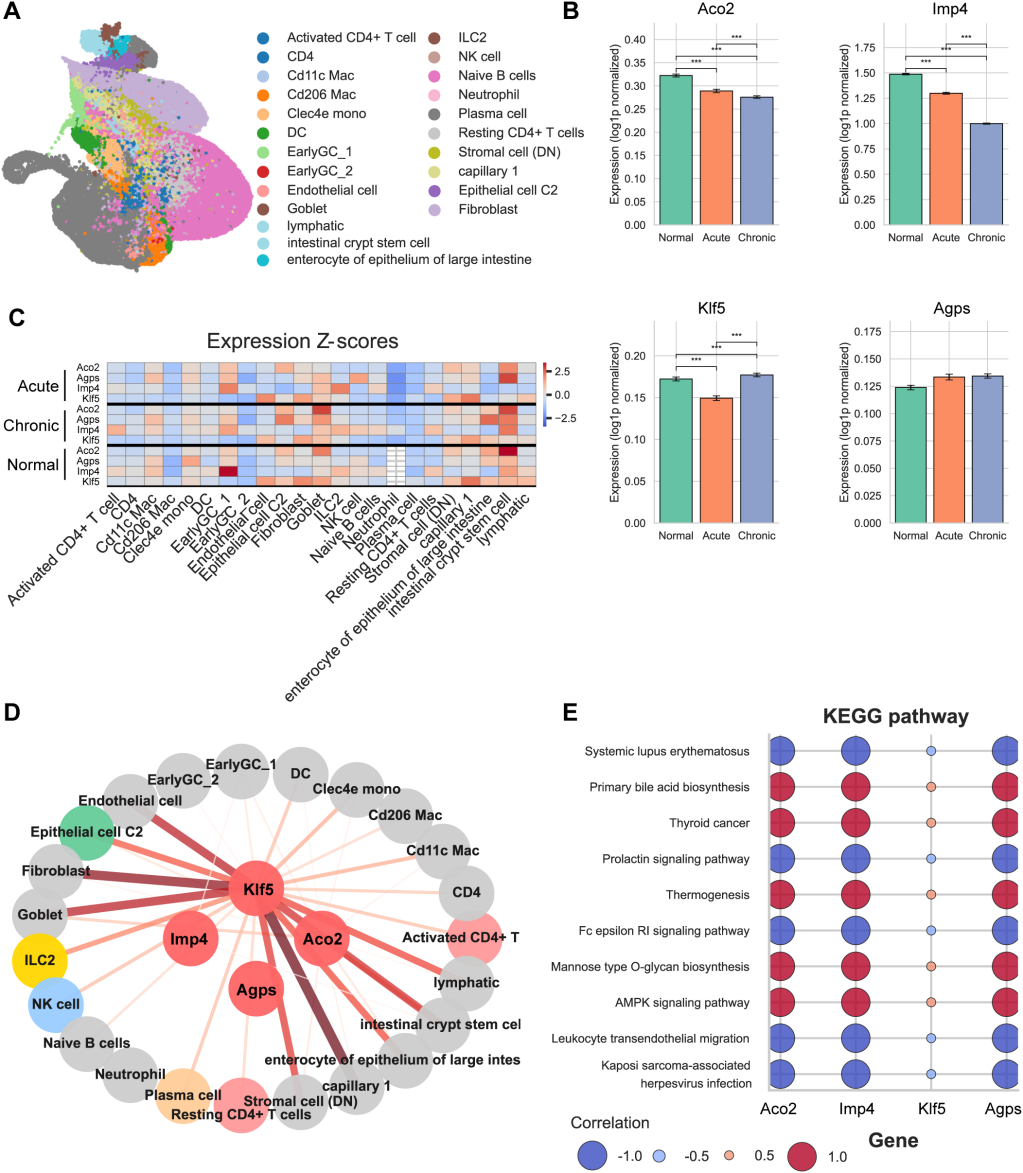
Single-cell validation of dynamic expression and functional associations of four key genes in a murine DSS-induced colitis model. **(A)** UMAP visualization showing distinct epithelial and immune cell clusters under normal, acute, and chronic colitis conditions. **(B)** Bar plots showing the average gene expression levels across disease stages based on all immune cell clusters combined. **(C)** Heatmap illustrating cell-type–specific expression dynamics of the four genes in different colitis stages. **(D)** Correlation network of the four genes (center nodes) with immune and stromal cell types (peripheral nodes). Edge color and thickness indicate correlation direction and strength. **(E)** KEGG enrichment bubble plot showing major immune- and metabolism-related pathways enriched for the four genes. (***p < 0.001).

To further validate the transcriptional dynamics of the four key genes in the context of inflammation, we analyzed single-cell RNA sequencing data from a murine dextran sulfate sodium (DSS)–induced colitis model (GSE264408). UMAP visualization revealed distinct clustering of epithelial and immune cell populations in the murine colon under normal, acute, and chronic colitis conditions (**Figure 8A**). The overall cell composition reflected disease progression, with immune cell expansion and epithelial remodeling in both acute and chronic inflammation.

Bar plot analysis based on all immune cells combined showed that Aco2 and Klf5 expression levels were significantly reduced in acute colitis and further decreased in the chronic stage, suggesting progressive transcriptional suppression during inflammation (**Figure 8B**). In contrast, Imp4 expression declined during the acute phase but was markedly upregulated in chronic colitis, indicating a possible compensatory or adaptive response in prolonged inflammation. Agps expression exhibited no significant difference among the three groups, consistent with its stable metabolic role observed in human data.

Heatmap visualization highlighted cell-type–specific expression patterns of these genes across epithelial and immune subsets (**Figure 8C**), further supporting their dynamic and compartmentalized regulation in colitis. Network analysis illustrated the correlation structure between the four key genes and various immune or stromal cell types (**Figure 8D**). The color and edge thickness represent the direction and strength of correlations, respectively. Notably, Klf5 showed predominant associations with epithelial and immune cell compartments, suggesting that its transcriptional activity may bridge epithelial integrity and immune regulation during colitis. Finally, KEGG pathway enrichment analysis (**Figure 8E**) demonstrated that these genes were mainly involved in immune–metabolic pathways, including the AMPK signaling pathway, leukocyte transendothelial migration, and thermogenesis.

### 3.8 Validation of hub gene expression in the DSS-induced murine colitis model

**Figure 9:**
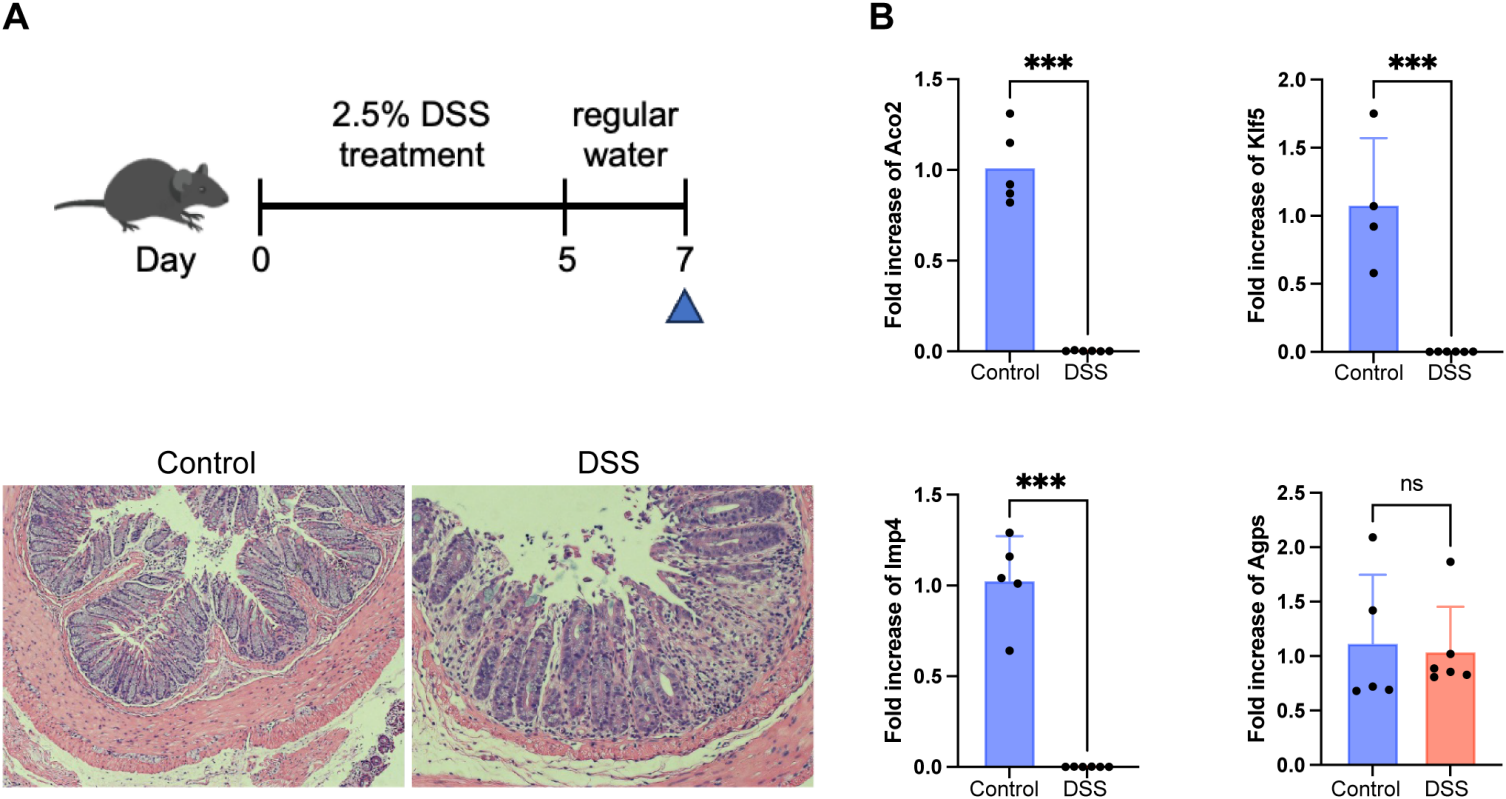
Validation of hub gene expression in the DSS-induced murine colitis model. **(A)** Schematic illustration of the experimental design for establishing the DSS-induced colitis model, and representative immunohistochemistry (IHC) images of mouse colon tissues. **(B)** Quantitative PCR analysis of Aco2, Klf5, Imp4, and Agps mRNA levels in colonic tissues from control and DSS-treated mice (n = 4-6). Expression of Aco2, Klf5, and Imp4 was significantly reduced in colitis samples, whereas Agps showed no significant difference. Data are presented as mean ± SD; p < 0.05 by Student’s t-test. (***p < 0.001).

To experimentally validate the transcriptional alterations of the four hub genes observed in human datasets, we established a DSS-induced murine model. IHC analysis revealed marked mucosal damage and epithelial disruption in DSS-treated mice compared with controls, confirming successful establishment of the colitis model (**Figure 9A**). qPCR analysis of colonic tissues revealed that Aco2, Klf5, and Imp4 expression levels were significantly decreased in DSS-treated mice compared with controls (**Figure 9B**). Gapdh was used as the internal control after confirming its stable expression between control and DSS-treated mice (unpaired t-test, p = 0.2264; **Supplementary Figure 1**). In contrast, Agps showed no significant change, consistent with its stable expression pattern observed in both bulk and single-cell transcriptomic analyses.

## 4 Discussion

Ulcerative colitis (UC) is a chronic inflammatory disorder of the colon characterized by epithelial barrier disruption, dysregulated immune responses, and metabolic imbalance. Despite considerable progress in understanding its pathogenesis, the precise molecular mechanisms linking immune dysregulation and cellular metabolism remain incompletely defined. In this study, we employed an integrative multi-omics framework combining MR, SMR, transcriptomic validation, and machine learning to identify candidate genes with causal and diagnostic relevance to UC. Through this comprehensive approach, we prioritized four hub genes — ACO2, KLF5, IMP4, and AGPS — supported by genetic and computational analyses, among which ACO2, KLF5, and IMP4 showed consistent transcriptomic validation. Subsequent immune infiltration profiling, scRNA-seq validation, and murine DSS colitis modeling further substantiated the functional relevance of the prioritized genes. Specifically, our findings highlight a mechanistic link between mitochondrial metabolism, epithelial remodeling, and immune activation, providing new insights into the immune–metabolic crosstalk underlying UC progression. These results not only extend previous genetic studies of UC but also underscore the value of integrating causal inference with transcriptomic and experimental validation to pinpoint functionally relevant targets for inflammatory bowel disease.

Among the identified hub genes, Aconitase 2 (ACO2) emerged as a central metabolic regulator linking mitochondrial dysfunction with mucosal inflammation. ACO2 encodes mitochondrial aconitase, a key enzyme of the tricarboxylic acid (TCA) cycle that catalyzes the interconversion of citrate and isocitrate (29). In our study, ACO2 was consistently downregulated in UC tissues and inflamed colonic regions, accompanied by enrichment of oxidative phosphorylation pathways, suggesting impaired mitochondrial bioenergetics in UC. Functional assays demonstrated that ACO2 loss suppresses mitochondrial oxidative phosphorylation while enhancing glycolysis and citrate flux, thereby fueling lipid biosynthesis and promoting tumor growth through upregulation of stearoyl-CoA desaturase (SCD1) (30). Another study identified ACO2 as part of a TCA cycle–related gene signature predictive of CRC survival, emphasizing its importance in metabolic homeostasis and disease progression (31). These results highlight ACO2 as a potential bridge between chronic inflammation and metabolic remodeling in the colonic mucosa, and a promising target for therapeutic interventions aimed at restoring mitochondrial homeostasis.

KLF5 (Kruppel-like factor 5) is a zinc-finger transcription factor involved in epithelial renewal, inflammation, and cellular proliferation. KLF5 is abundantly expressed in proliferating crypt cells and is rapidly induced following epithelial injury or stress stimuli (32). Loss-of-function studies have shown that Klf5^+/–^ mice exhibit increased susceptibility to DSS-induced colitis and impaired mucosal recovery, primarily due to reduced epithelial proliferation and migration (33). Conversely, epithelial-specific Klf5 overexpression alleviates DSS-induced colonic injury by activating the JAK–STAT pathway and promoting epithelial repair (34). Furthermore, Klf5 is required for the self-renewal and lineage specification of intestinal stem cells through epigenetic regulation of WNT- and NOTCH-responsive genes; its absence leads to premature differentiation and defective epithelial regeneration (35). Collectively, these findings suggest a critical role for KLF5 in maintaining epithelial integrity and repair, consistent with its downregulation observed in UC tissues.

Beyond its epithelial functions, KLF5 also modulates immune responses in the inflamed gut. Epithelial-specific Klf5 deletion leads to Th17-dependent colonic inflammation accompanied by aberrant STAT3 activation and dysregulated IL-22 signaling, implicating KLF5 in suppressing excessive Th17 responses (36). In our study, the upregulation of KLF5 in CD4^+^ activated FOS-hi and CD8^+^ LP T-cell subsets suggests that KLF5 may also act as a transcriptional activator within effector T cells, potentially contributing to chronic immune activation. This cell-type–specific upregulation, together with the positive causal association identified by MR/SMR analysis, supports a pro-inflammatory role of KLF5 in adaptive immunity. Notably, although KLF5 and ACO2 were downregulated in UC, they positively correlated with immune cell infiltration. This likely reflects differences between group-level comparisons and within-cohort variability in a heterogeneous disease context, suggesting context-dependent associations rather than direct causality.

IMP4 (U3 small nucleolar ribonucleoprotein protein 4 homolog) is a highly conserved nucleolar factor that participates in ribosome biogenesis, cell-cycle regulation, and oncogenesis. Functionally, IMP4 forms a core trimeric complex with Mpp10 and Imp3, which is essential for the assembly of the small subunit (SSU) process some and early pre-rRNA processing (37). The nucleolar protein Sas10 directs the delivery of this Mpp10-Imp3-Imp4 complex to the nucleolus and stabilizes Mpp10 against proteolytic degradation, underscoring IMP4’s critical structural role in ribosome formation and organ development (37).Beyond its canonical role in ribosome biogenesis, transcriptomic profiling has revealed that IMP4 expression is tightly coupled to ribosomal and proliferative signaling pathways. In colorectal cancer, IMP4 has emerged as a ribosome-associated oncogene. Weighted gene co-expression and PPI network analyses identified IMP4 as a hub gene in CRC-enriched modules related to ribosomal protein synthesis and lncRNA-associated oncogenic networks (38). Its expression was significantly elevated in tumor tissues compared to adjacent normal mucosa and strongly correlated with genes involved in Myc targets, oxidative phosphorylation, and cell proliferation (39). High IMP4 expression was also associated with unfavorable prognosis and potential drug resistance, suggesting its dual diagnostic and therapeutic relevance (39). Although IMP4 has not been extensively characterized in inflammatory bowel disease, its molecular function implies a potential connection with mucosal regeneration and inflammatory metabolism. Given that ribosome biogenesis and translational reprogramming are tightly linked to epithelial renewal and immune activation in ulcerative colitis (40), dysregulation of IMP4 may contribute to aberrant epithelial proliferation or stress-induced translational responses in inflamed mucosa. Integrating these findings, IMP4 could act as a ribosome biogenesis–immune signaling bridge, whose overactivation promotes hyperproliferation and metabolic stress, while its inhibition might impair mucosal repair. Further experimental validation in intestinal models is warranted to delineate its precise role in UC pathogenesis.

Alkylglycerone phosphate synthase (AGPS) is a key enzyme involved in ether lipid and plasmalogen biosynthesis, influencing membrane dynamics, redox balance, and inflammatory signaling (41). Alterations in lipid metabolism have been increasingly recognized as a hallmark of UC, contributing to epithelial barrier dysfunction and dysregulated host–microbiota interactions (42). Although AGPS did not exhibit significant transcriptional changes in our datasets, it emerged as an important predictive feature in the machine learning model and demonstrated high network connectivity in the co-expression analysis. As a key enzyme involved in ether lipid biosynthesis, AGPS has established roles in lipid metabolism and membrane signaling. Therefore, we speculate that its potential contribution to UC may involve functional metabolic regulation rather than overt transcriptional alterations. Further mechanistic studies are required to clarify its role in immune–metabolic remodeling.

Despite the robust integrative approach and consistent findings across multiple datasets, several limitations of this study should be acknowledged. First, the analyses were primarily based on publicly available bulk and single-cell transcriptomic data, which, although cross-validated in both human and murine systems, remain correlative in nature. The causative roles of the identified hub genes—particularly their cell-type–specific regulatory effects—require further functional validation through loss- and gain-of-function experiments in relevant immune and epithelial cell subsets. Second, although the DSS model provides mechanistic insight, it only partially recapitulates human UC, and additional validation in patient-derived organoids or humanized models would strengthen the translational relevance. Third, although our integrative pipeline combined genetic colocalization, transcriptomic profiling, and pathway analysis, it did not fully capture post-transcriptional or epigenetic regulation, such as noncoding RNA networks and chromatin accessibility dynamics, which may also shape the immune–metabolic crosstalk observed here.

In summary, our integrative multi-omics framework identified ACO2, KLF5, and IMP4 as central regulators connecting mitochondrial metabolism with mucosal immune remodeling in UC. These genes showed consistent downregulation in human UC tissues, dynamic expression changes in murine colitis, and strong associations with immune and epithelial cell compartments. Together, these findings enhance our understanding of UC immunometabolism and highlight promising targets for therapeutic modulation.

## Data Availability

The datasets presented in this study can be found in online repositories. The names of the repository/repositories and accession number(s) can be found in the article/**Supplementary Material**.
