# Supplementary material for: Integrative multi-omics dissection identifies ACO2, KLF5, and IMP4 as central regulators of the mitochondrial–immune axis in ulcerative colitis
Source: Front Immunol. 2026 Mar 27;17:1746810. doi: 10.3389/fimmu.2026.1746810 (PMC13066277; doi:10.3389/fimmu.2026.1746810)
Supplement: Supplementary file 5 [file Table5.docx]

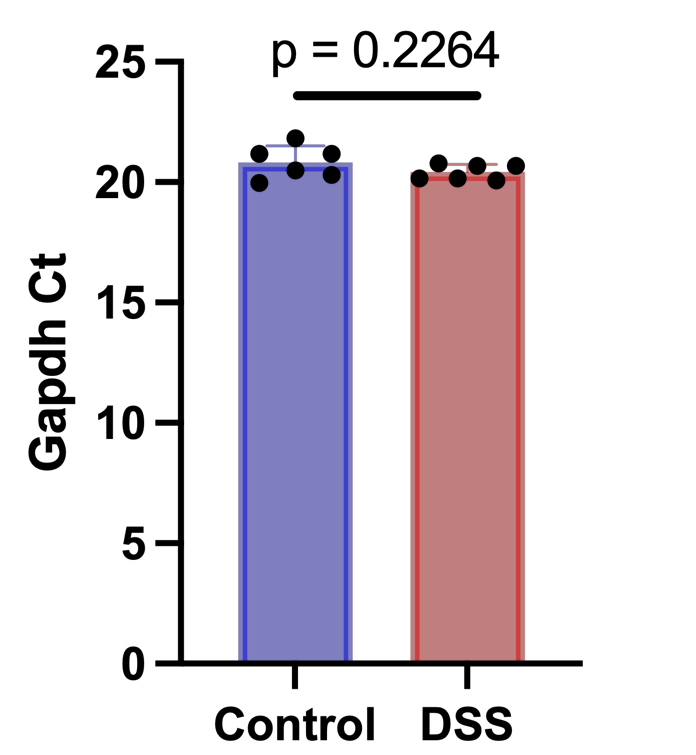


**Supplementary Figure 1. Stability analysis of Gapdh expression in the DSS-induced colitis model.** Raw Ct values of Gapdh in control and DSS-treated mice (2.5% DSS for 5 days followed by 2 days of regular water). No significant difference was observed between groups (unpaired two-tailed t-test: t = 1.289, df = 10, p = 0.2264). Data are presented as mean ± SD. n = 6 mice per group.
